# Quality of life following road traffic injury: the impact of age and gender

**DOI:** 10.1007/s11136-020-02427-3

**Published:** 2020-01-20

**Authors:** R. Rissanen, J. Ifver, M. Hasselberg, H.-Y. Berg

**Affiliations:** 1 Department of Global Public Health, Karolinska Institutet, Tomtebodavägen 18A, Widerströmska huset, 17177 Stockholm, Sweden; 2 Swedish Transport Agency, 781 23 Borlänge, Sweden

**Keywords:** Health Related Quality of Life, Gender, Road traffic injury, Injury severity, AIS

## Abstract

**Purpose:**

The impact of road traffic crashes on health is well developed, in terms of deaths and direct consequences, but it is less so in terms of long-term life consequences. Few studies have compared the general impact on Health Related Quality of Life (HRQoL) following road traffic injury (RTI) by using a variety of different injured body parts and severity levels of the injury and compared them with a sample of non-injured referent individuals. Consequently, the aim of the current study is to assess how injury severity is associated with HRQoL, and if it differs between men, women, over age and injured body parts.

**Methods:**

This cross-sectional study identified people with a RTI in the Swedish Traffic Accident Data Acquisition System (STRADA). A frequency matched reference group was also included. Data include both register data and self-reported HRQoL data.

**Results:**

A total of 1788 out of 4761 persons with an RTI (37.6%) and 2186 out of 4761 reference persons (45.9%) returned the questionnaire, giving a total response rate of 41.9% (*n* = 3974). The findings show different patterns of HRQoL loss, depending on sex, age, injured body part, and levels of injury severity.

**Conclusion:**

The results show that even relatively minor road traffic injuries can lead to a significantly lower of HRQoL, especially for women, compared to the non-injured reference group. Moreover, when the inherent reduction of HRQoL over age was considered, the results indicated that younger individuals have a larger difference from the reference group in HRQoL, independent of the injury severity, compared to the older individuals; hence, an improved understanding of age and gender differences in HRQoL following an RTI is needed to better understand the long-term consequences of injuries from a public health perspective.

**Electronic supplementary material:**

The online version of this article (10.1007/s11136-020-02427-3) contains supplementary material, which is available to authorized users.

## Introduction

Road traffic injuries cause major health loss in the world [1], and it is predicted to be the seventh leading cause of death in 2030 [2]. The available documentation describing the impact of road traffic crashes on health is well developed in terms of deaths and direct consequences, but it is less so in terms of long-term life consequences that arise as a result of road traffic crashes. Police and hospital data reflect the direct and short-term health consequences of an injury following a crash but are silent regarding the long-term consequences, as this type of data only captures the acute phase of the crash [3, 4]. The immediate outcome of a road traffic injury (RTI) might differ from the long-term outcomes. One way of summarizing the long-term outcome of an RTI is in terms of Health Related Quality of Life (HRQoL). HRQoL assessments are often subjective and self-reported and can incorporate multidimensional constructs, including physical health, psychological state, level of independence, social relationships, and relationship to salient features of their environment [5]. The overall loss of HRQoL is known to be detrimental and long lasting for persons injured in road traffic crashes, and both physical and psychological consequences are considerable for those affected [6]. It is important to note that it is not only the more severe injuries that are negatively associated with lower HRQoL following RTI; rather, all injury severity levels (minor, moderate, and severe injuries) have been shown to have a negative effect on HRQoL [7–11]. For example, a qualitative Swedish study [12] indicated that individuals who suffered minor and moderate injuries following a road traffic crash (severe injuries not included) reported long-term life consequences. These consequences included physical, psychological, financial, and everyday life consequences. A surprising finding of the study was that women and men report different consequences following RTI in relation to the psychological and everyday life consequences. These reported differences included psychological reactions like travel anxiety and PTSD-like symptoms, which were reported by the women but not the men.

These results are supported by the findings of Monárrez-Espino et al. [13] who used the same population as in the current study to compute a composite score, which takes into account the key injury dimensions (i.e., number of body parts affected, location, and severity of injury) to investigate whether the composite score was predictive of the risk of low quality of life (QoL) (< 90% of the non-injured referents). Their main results showed that compared with non-injured individuals, road traffic crash victims of any injury category had a higher risk, expressed as odds ratio, of lower QoL 1 to 4 years after the crash; however, the probability of lower QoL was increased substantially with a higher composite score [13]. Although a higher risk was detected for those injured, the study did not regard if injuries to specific body regions would increase the risk of lower HRQoL, as the composite score regarded only the number of injuries and not injuries to specific body parts or regions. Few studies have used a variety of different injured body parts (e.g., head, neck, lower extremity) and different severity levels of the injury and compared them with a sample of non-injured referent individuals in one study [6].

Moreover, although several studies have investigated the consequences of RTI in terms of HRQoL, few studies have, to our knowledge, considered the inherent loss of HRQoL over age and gender in their analysis [6]. Rather, these studies have compared the loss of HRQoL in an injured population to a constant HRQoL level in a referent group, and the inherent loss of HRQoL over age has not been considered in these studies. Hence, there is a lack of detailed knowledge on the comparison of different injured body parts and severities across different genders and ages, in relation to age specific HRQoL. Consequently, the aim of the current study was to assess how injury severity was associated with HRQoL, and if it differed between men, women, over age, and injured body parts and to compare the HRQoL of people suffering an RTI to a referent population.

## Materials and methods

To assess the HRQoL following RTI, this cross-sectional study used a mixed mode design in the data collection, including both register-based data and self-reported questionnaires. Individuals with an RTI were identified in the Swedish Traffic Accident Data Acquisition System (STRADA). They were asked to fill out a self-reported questionnaire. A frequency matched reference group, i.e., a group of controls are matched to a group of cases, was sampled from the Swedish Total Population Register.

### Health Related Quality of Life

The self-reported questionnaire included an assessment of HRQoL by EQ-5D [14]. EQ-5D is a standardized measure of self-rated health, which assesses HRQoL in five dimensions: mobility, self-care, usual activities, pain/discomfort, and anxiety/depression. Each dimension has three levels: no problems, some problems, and extreme problems. The indicated health state can be converted into a single summary index, which ranges from full health indicated by 1 to a health status worse than dead by applying a weight to each of the levels in each dimension [14]. For the current study, we used the coefficients for EQ-5D health states, based on the UK population health survey [15–17]. Time trade-off value sets for the health states were as follows: Full health for all five dimensions = 1; mobility 2 =  − 0.069 and 3 =  − 0.314; self-care 2 =  − 0.104 and 3 =  − 0.214; usual activities 2 =  − 0.036 and 3 =  − 0.094; pain/discomfort 2 =  − 0.123 and 3 =  − 0.386; anxiety/depression 2 =  − 0.071 and 3 =  − 0.236; and constants when at least one with 2 or 3 =  − 0.081 and when at least one with 3 =  − 0.269. For example, for a health state of 21,232, the score would be 0.088 [1– (0.069 + 0 + 0.036 + 0.386 + 0.071 + 0.081 + 0.269)] [18].

### Injury severity and injured body part

Injury data, including injured body part and its corresponding injury severity were classified by the Abbreviated Injury Scale (AIS), derived from STRADA. The AIS score represents the probability of death associated with a single injury [19]. To assess the injury severity, we used the Maximum AIS score (MAIS) [20]. The MAIS addresses multiple injuries and is based on the AIS [19] of the most severe injury. Severity was defined as injuries assessed as MAIS1 to MAIS5, according to the MAIS six graded scale, where the sixth grade represents injuries that are not survivable. If a person had two or more equal MAIS-values, one of the injured body parts was randomly selected.

### Participants

A stratified sample of individuals injured in traffic in Sweden between January 2007 and December 2009 was identified in STRADA in 2010. STRADA contains nationally collected injury data, which are reported to the system both by the police and the emergency care hospitals [21]. It includes RTI data such as severity and injured body part [21], which were used to stratify the sample based on sex, injured body part, and its corresponding AIS-value. Participants were further categorized by using the MAIS [20] on one injured body part. In order to gain representation of different injured body parts and statistical power, we aimed to include 5000 individuals based on ten body parts and five MAIS-values (MAIS1-5). The body parts included: head, cervical spine, face, upper extremities, lower extremities and pelvis, thorax, thoracic spine, abdomen, lumbar spine, and external. We aimed to include 100 individuals per body part and injury severity classification. However, it was noted during the sampling that several body parts and injury severities did not fulfill the expected 100 observations (e.g., minor MAIS for abdomen) and some body parts and injury severities had more observations than the expected 100. Hence, 100 individuals were randomly selected for those categories that contained more than 100 individuals in total; for the categories that included less than 100 individuals, all individuals were included in the final sample.

A reference group was selected from the Swedish Total Population Register in August 2010. Frequency matching was done by age (i.e., month and year of birth) and sex.

In November 2010, an informational letter about the study and an invitation to participate, together with a short questionnaire were sent, both to participants with injuries and the reference group. For participants under the age of 15, a written consent form from one of the guardians was required for participation. If participants had not returned their questionnaire after 3 weeks, a first reminder was sent; thereafter, a second reminder was sent after 6 weeks. Of the 4761 persons with injuries, a total of 1884 persons (39.5%) returned the questionnaire, out of which 96 individuals were excluded due to reporting a previous injury or having a disability. Out of the 4761 reference individuals from the Swedish Total Population Register, a total of 2263 (47.5%) returned the questionnaire in full, out of which 77 individuals were excluded due to reporting a previous injury or having a disability, giving a total inclusion rate of 41.9% (*n* = 3974).

The average age was 46.2 for the participants with injuries and 46.8 for the reference group. There were some differences between those who responded and those who did not (see Table 1 for details). There were significantly more females (59%) among the respondents compared to the non-respondents, in the reference group among the middle aged (70%) and elderly respondents (64%). Furthermore, the response rate for those with MAIS1 classified injuries to the head, lumbar spine, and external injuries was significantly lower compared to individuals with MAIS1 classified injuries to other body parts (data not shown). Concerning the MAIS2 classified injuries, there were more respondents with injuries to the cervical and lumbar spine and upper extremities who responded to the questionnaire than other body parts with MAIS2 classified injuries (see Online Appendix). No significant differences were detected regarding the MAIS3+ category injuries regarding the respondents and the non-respondents.

**Table 1:** Characteristics of injured and referent persons, by gender, age group, and responders and non-responders

Injured and non-injured			Gender						Total		
			Male			Female					
			Responding *n* (%)		Total *n* (%)	Responding *n* (%)		Total *n* (%)	Responding *n* (%)		Total *n* (%)
			No	Yes		No	Yes		No	Yes	
Referents	Age	7–17	321 (81.1)	75 (18.9)	396 (100)	171 (63.8)	97 (36.2)	268 (100)	492 (74.1)	172 (25.9)	664 (100)
		18–29	526 (76.2)	164 (23.8)	690 (100)	218 (55.1)	178 (44.9)	396 (100)	744 (68.5)	342 (31.5)	1086 (100)
		30–64	654 (52.4)	594 (47.6)	1248 (100)	264 (29.8)	621 (70.2)	885 (100)	918 (43.0)	1215 (57.0)	2133 (100)
		65–	201 (52.2)	184 (47.8)	385 (100)	151 (35.6)	273 (64.4)	424 (100)	352 (43.5)	457 (56.5)	809 (100)
	Total		1702 (62.6)	1017 (37.4)	2719 (100)	804 (40.8)	1169 (59.2)	1973 (100)	2506 (53.4)	2186 (46.6)	4692 (100)
Injured	Age	7–17	290 (73.2)	106 (26.8)	396 (100)	176 (65.9)	91 (34.1)	267 (100)	466 (70.3)	197 (29.7)	663 (100)
		18–29	532 (77.1)	158 (22.9)	690 (100)	241 (60.9)	155 (39.1)	396 (100)	773 (71.2)	313 (28.8)	1086 (100)
		30–64	796 (63.8)	452 (36.2)	1248 (100)	450 (50.8)	435 (49.2)	885 (100)	1246 (58.4)	887 (41.6)	2133 (100)
		65–	206 (53.5)	179 (46.5)	385 (100)	212 (50.0)	212 (50.0)	424 (100)	418 (51.7)	391 (48.3)	809 (100)
	Total		1824 (67.1)	895 (32.9)	2719 (100)	1079 (54.7)	893 (45.3)	1972 (100)	2903 (61.9)	1788 (38.1)	4691 (100)
Total population	Age	7–17	611 (77.1)	181 (22.9)	792 (100)	347 (64.9)	188 (35.1)	535 (100)	958 (72.2)	369 (27.8)	1327 (100)
		18–29	1058 (76.7)	322 (23.3)	1380 (100)	459 (58.0)	333 (42.0)	792 (100)	1517 (69.8)	655 (30.2)	2172 (100)
		30–64	1450 (58.1)	1046 (41.9)	2496 (100)	714 (40.3)	1056 (59.7)	1770 (100)	2164 (50.7)	2102 (49.3)	4266 (100)
		65–	407 (52.9)	363 (47.1)	770 (100)	363 (42.8)	485 (57.2)	848 (100)	770 (47.6)	848 (52.4)	1618 (100)
	Total		3526 (64.8)	1912 (35.2)	5438 (100)	1883 (47.7)	2062 (52.3)	3945 (100)	5409 (57.6)	3974 (42.4)	9383 (100)

### Statistical analyses

Due to a small number of injured individuals in the higher MAIS-levels, the MAIS3, 4, and 5 were merged into a new category called MAIS3+ . Moreover, when studying the number of responses, it was noted that there were very few responses from the youngest children (0–6 years); therefore, this age group was excluded from the analysis. Due to skewness of the data in the dependent HRQoL-variable, a non-parametric test was used, i.e., a Kruskal–Wallis and Mann–Whitney tests. Because of the risk of a problem with mass significance, a risk level of *p* = 0.017 was selected based on the Bonferroni correction, instead of the conventional risk level of *p* = 0.05. The IBM SPSS Statistics v. 22 was used to perform the calculations.

This project has been approved by the Regional Ethical Review Board in Stockholm (protocol 2009/5:12).

## Results

The group with injuries (total) reported significantly lower HRQoL index scores, irrespective of the MAIS category, compared to their non-injured counterparts in the reference group (*p* < 0.017). When the MAIS categorization was considered in the analysis, the results indicated that participants with the MAIS3 classified injuries reported significantly lower HRQoL compared to the two other MAIS classifications (see Table 2).

**Table 2:** EQ-5D index divided by age, sex, and MAIS

					EQ-%D index by MAIS					
	Reference		Injured		MAIS1		MAIS2		MAIS3	
	Mean (± CI)	*n*	Mean (± CI)	*n*	Mean (± CI)	*n*	Mean (± CI)	*n*	Mean (± CI)	*n*
Total	0.878 (± 0.012)	2186	0.796^a^ (± 0.019)	1788	0.842 (± 0.030)	590	0.806 (± 0.028)	752	0.718^c^ (± 0.043)	446
Age group										
7–17	0.943 (± 0.025)	172	0.847^a^ (± 0.051)	197	0.890 (± 0.066)	78	0.863 (± 0.075)	72	0.752^c^ (± 0.132)	47
18–29	0.913 (± 0.023)	342	0.788^a^ (± 0.049)	313	0.830 (± 0.067)	141	0.781 (± 0.081)	114	0.698^b^ (± 0.129)	58
30–64	0.885(± 0.016)	1215	0.797^a^ (± 0.027)	887	0.837 (± 0.042)	296	0.805 (± 0.040)	391	0.724^c^ (± 0.067)	200
65–	0.810 (± 0.032)	457	0.773^a^ (± 0.040)	391	0.833 (± 0.077)	75	0.801 (± 0.055)	175	0.706^c^ (± 0.072)	141
Female										
7–17	0.951 (± 0.030)	97	0.788^a^ (± 0.040)	91	0.852(± 0.107)	42	0.823 (± 0.132)	26	0.632^b^ (± 0.222)	23
18–29	0.928(± 0.029)	178	0.774^a^ (± 0.069)	155	0.805 (± 0.090)	75	0.770 (± 0.116)	60	0.669^c^ (± 0.214)	20
30–64	0.904 (± 0.020)	621	0.777^a^ (± 0.041)	435	0.817 (± 0.057)	162	0.779 (± 0.061)	195	0.690^b^ (± 0.117)	78
65–	0.836 (± 0.036)	273	0.742^a^ (± 0.058)	212	0.790 (± 0.113)	45	0.772 (± 0.085)	92	0.676^c^ (± 0.104)	75
Male										
7–17	0.943 (± 0.044)	75	0.898 (± 0.050)	106	0.935 (± 0.063)	36	0.885 (± 0.089)	46	0.868 (± 0.102)	24
18–29	0.896 (± 0.036)	164	0.801^a^ (± 0.070)	158	0.859 (± 0.099)	66	0.794 (± 0.115)	54	0.713^b^ (± 0.163)	38
30–64	0.865 (± 0.025)	594	0.817^a^ (± 0.037)	452	0.860 (± 0.062)	134	0.830 (± 0.052)	196	0.746^c^ (± 0.079)	122
65–	0.771 (± 0.059)	184	0.809 (± 0.051)	179	0.898 (± 0.080)	30	0.832 (± 0.066)	83	0.740^c^ (± 0.098)	66
Sex										
Male	0.858 (± 0.020)	1017	0.822 (± 0.025)	895	0.874 (± 0.042)	266	0.832 (± 0.036)	379	0.751^d^ (± 0.054)	250
Female	0.896 (± 0.015)	1169	0.769^a^ (± 0.028)	893	0.815 (± 0.041)	324	0.779 (± 0.043)	373	0.676^c^ (± 0.070)	196

^a^Indicates a statistically significant difference between the reference group and the injury group

^b^Indicates a statistically significant difference between MAIS1 and 3

^c^Indicates a statistically significant difference between MAIS1 and 3, and MAIS2 and 3

^d^Indicates a statistically significant difference between all of the MAIS groups

When analyzing HRQoL according to age, the results showed that there is a significant difference in the HRQoL index scores across all the different age groups with MAIS1 classified injuries reporting the highest HRQoL. When dividing the age groups based on sex, the results indicated that all of the age groups, except for males aged 7–17, displayed significant differences regarding HRQoL based on the MAIS classification (see Table 2 for details regarding the significance).

**Fig. 1:**
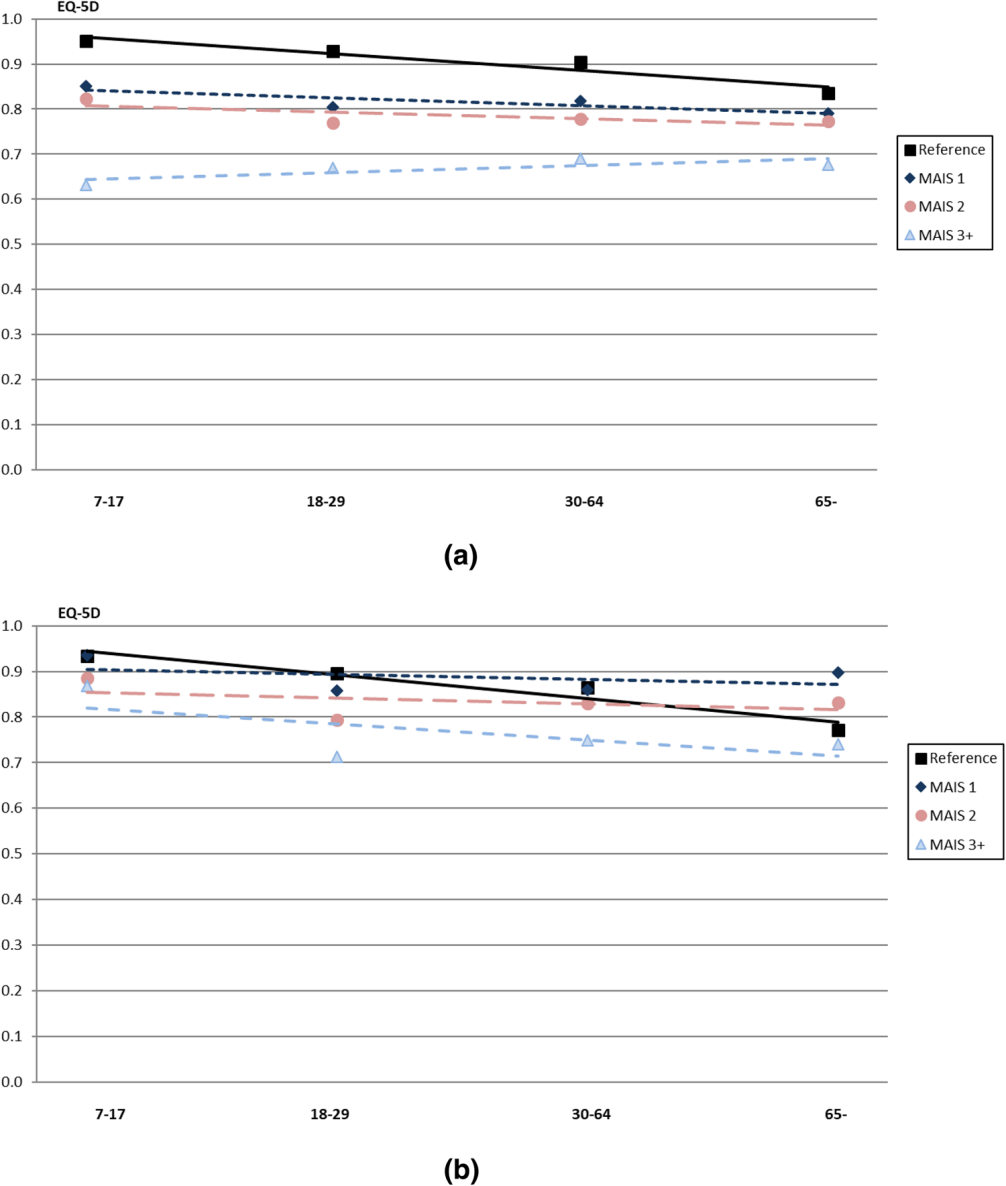
**a** HRQoL by MAIS and age for *females*, compared to the reference group, **b** HRQoL by MAIS and age for *males*, compared to the reference group

When the group with individuals who were injured was divided by sex, the results showed that females reported a lower HRQoL index score compared to the males (0.769 vs. 0.822, respectively) (Table 2). On average, women had a higher HRQoL than men in the reference group (0.896 vs. 0.858), while the opposite was true for the women with injuries in the three MAIS groups (MAIS1, MAIS2, and MAIS3+), who reported statistically lower HRQoL compared to the men with injuries (0.815 vs. 0.874, 0.779 vs. 0.832, 0.676 vs. 0.751, respectively). Men with injuries reported a HRQoL loss within all age groups for the MAIS3+ injuries and among younger age groups within MAIS2 and to a certain degree MAIS1 injuries, whilst the women with injuries reported a HRQoL loss in all age groups and all MAIS categories. Younger women with injuries reported a lower HRQoL index score compared to older females. There was a clear difference in all age groups between MAIS1, MAIS2, and especially MAIS3+ injuries compared to the reference group. However, the difference between the reference group and the three different MAIS groups became lesser in the older age groups. Interestingly, men, on the other hand, in the age group of 30 and over (MAIS1 and 2) reported a HRQoL index score equal to or even higher compared with the reference group (Fig. 1a, b).

### HRQoL by injured body part and MAIS

**Fig. 2:**
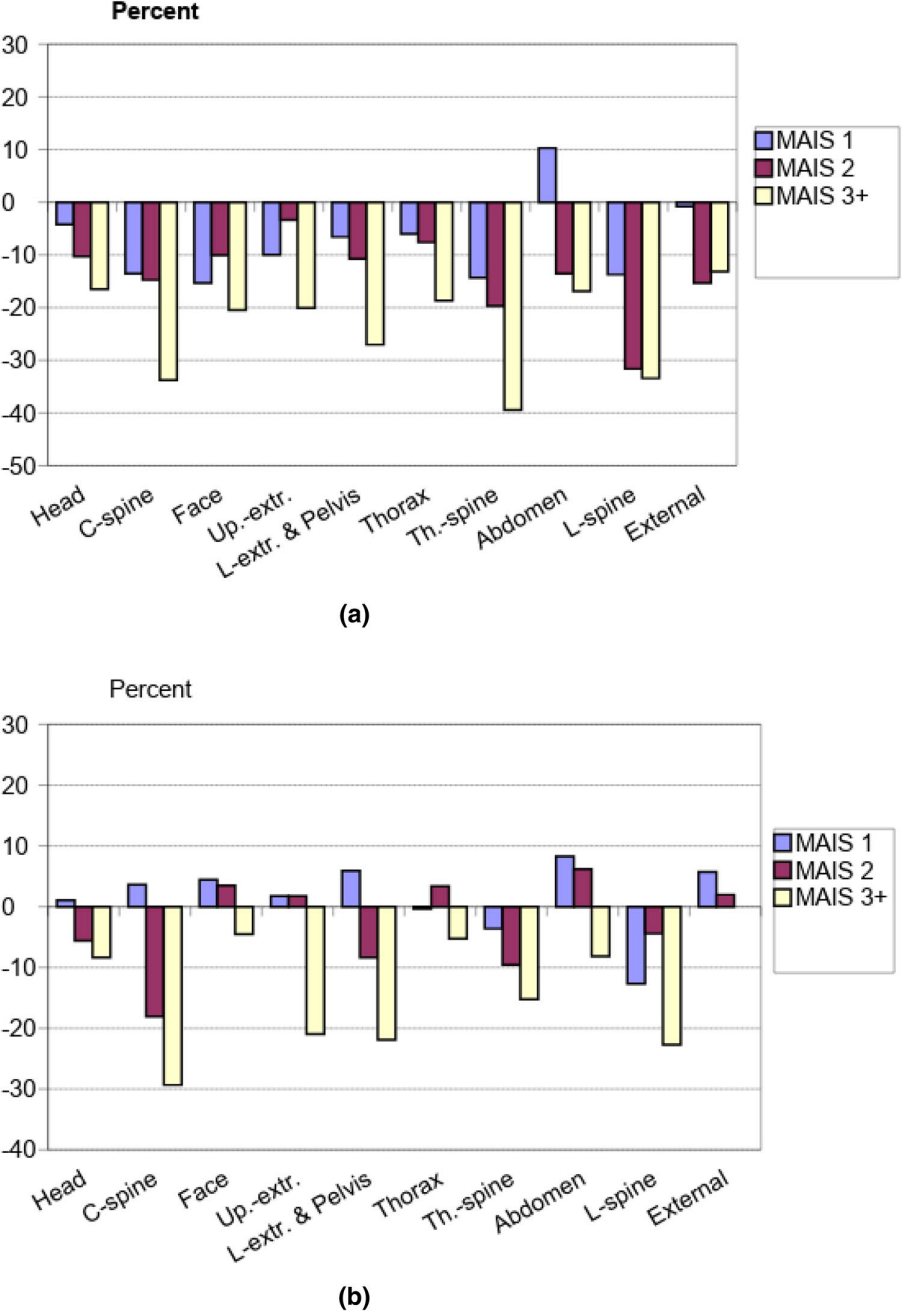
**a** Percental difference in HRQoL, divided by MAIS and injured body part for *females* compared to the reference group, represented by 0%, **b** Percental difference in HRQoL, divided by MAIS and injured body part for *males* compared to the reference group, represented by 0%

The lowest HRQoL was observed for those with the highest MAIS score. MAIS3 + injuries to the “Cervical Spine,” “Lumbar Spine,” “Thoracic Spine,” “Lower Extremities and Pelvis,” and “Upper extremities” disclosed the highest percental loss of HRQoL in comparison to the reference group. Moreover, injuries classified as MAIS2 also resulted in a higher percental difference in HRQoL, especially the MAIS2 injuries on “Lumbar Spine,” “Thoracic Spine,” and “Cervical Spine.” Although the highest percental difference in HRQoL was observed in the higher injury grades, injuries classified as MAIS1 might have caused a higher percental difference in HRQoL compared to a higher grade of MAIS. For example, MAIS1 injuries to the “Lumbar Spine” had a 14% difference in HRQoL, whilst a MAIS3+ injury on the “Thorax” had a 11% difference in HRQoL. See Fig. 2a, b for HRQoL divided by MAIS and injured body part for the total study population.

### HRQoL by sex, injured body part and MAIS

When the HRQoL loss was analyzed in relation to sex, the results indicated that although women showed a similar pattern as the total study population in relation to the difference in HRQoL and injury severity, i.e., a higher MAIS score indicated a larger percental difference in HRQoL; women also displayed a substantially lower HRQoL for the minor injuries classified as MAIS1, except for injuries to the abdomen (Fig. 2a). This was especially prominent when analyzing women’s HRQoL following MAIS1 classified injuries to the face and upper extremities, which displayed a lower reported HRQoL for the MAIS1 injuries compared to the MAIS2 and MAIS3 classified injuries for the same body parts. The MAIS1 classified injuries did not result in a substantially lower HRQoL for the men as it did for the women; instead, men reported a HRQoL that was lower in relation to MAIS2 and MAIS3 classified injuries, except for injuries on the thoracic- and lumbar spine, see Fig. 2a, b.

## Discussion

The results from this study show that even relatively minor road traffic injuries can lead to a significantly lower HRQoL, especially among women. The findings show different patterns of HRQoL after RTI, depending on sex, age, injured body part, and levels of injury severity. It was found that all degrees of injury severity (MAIS classification) led to a significantly lower HRQoL for women, primarily in the younger age groups, compared to the reference group. However, for men, this difference in HRQoL was found only for more severe injuries (MAIS3+). These results confirm findings from other studies, where men reported significantly better HRQoL after an RTI than women. The results also confirmed that the HRQoL generally decreases with higher injury severity [6, 9, 22] and that women do not recover their HRQoL in the same way as men [9, 10, 22, 23]. However, this study adds additional knowledge since we had the possibility to assess the natural reduction of HRQoL by using a reference group. If this natural reduction is not considered in the comparison of HRQoL of those with injuries, the results indicate that older people with injuries are more negatively affected in relation to HRQoL, in all injury severities, compared to their younger counterparts. However, if the reference group’s reduction of HRQoL over age is considered in the analysis, then the interpretation becomes the opposite. Hence, younger individuals have a larger difference from the reference group in HRQoL, independent of the injury severity, compared to the older individuals, due to the reduction of HRQoL with increased age, since the level of HRQoL is already reduced for the older individuals. In other words, the difference between the injured group and the reference group was reduced with increased age, but women showed a lower HRQoL compared to men (Table 2), regardless of age and injury severity. Women in the older age groups had not recovered to the reference group’s level regardless of the injury severity, while men in the age group of 30–64 years with less severe injuries (MAIS1 and 2) (MAIS1) and men in the age group of 65 years with injuries classified as MAIS2 did reach the same level as the reference group. However, participants with more severe injuries (MAIS3+) did not reach the reference group’s HRQoL levels, regardless of age and sex. One conclusion is therefore that all levels of injury severity lead to a lower HRQoL for women, primarily in the younger age groups, whilst only more severe injuries (MAIS3+) lead to a lower HRQoL among men. Whether women suffer more from the psychological effects of RTI or if women tend to communicate about their injuries in different ways than men need to be further investigated. A qualitative study from our research group indicated that men and women report similarities regarding the experienced consequences of an injury, but they also report different consequences, both considering the type and the severity [6]. There is evidence that women have worse physical outcomes after an injury due to their smaller body structure [24]; however, more studies are needed to determine if the difference in outcome can be explained by differences in crash mechanism, from treatment variables, or from premorbid sex differences.

Not surprisingly, the results show that more severe injuries (MAIS3+) gave a substantially lower HRQoL in general, but also that it is related to gender and injured body part. Furthermore, the results show that even less severe injuries (MAIS1) can give considerably lower HRQoL, especially for women (see Fig. 2a, b for details on body parts). In accordance with previous studies, injuries to the cervical-, thoracic and lumbar spine, upper- and lower extremities, and pelvis result in significantly lower HRQoL [6]. For the MAIS2 classified injuries, the low HRQoL was most substantial in relation to injuries to the cervical-, thoracic, and lumbar spine. For the MAIS1 classified injuries, the low HRQoL was most common in relation to injuries to the lumbar- and thoracic spine with a negative difference of 10–14% for the injury group compared to the reference group. In line with previous studies, the results show that injuries to the spine have the most impact on HRQoL, independent of the injury severity [9, 11, 25].

### Strengths and limitation

The main strengths of the study are that it includes a nationwide sample of RTIs and the comparison of injury severity and injured body parts, which gives an estimation of the impact of the different injuries and injury severities on HRQoL, both in total and for each gender, specifically in relation to a reference group without known injuries. However, some limitations also need to be mentioned in relation to this study, mainly arising from the low response rates for those with injuries and the reference group. Due to the low response rate, an extensive analysis was performed to identify possible biases. The analysis did not show a clear and consistent pattern of non-participation between gender and age groups, or between the injury group and the reference group. Both the injury group and the reference group followed similar non-response patterns, which suggests that major differential bias between the two groups is unlikely. We have also recognized the lower participation of younger individuals, especially men (who are healthier than older men); however, this pattern is common in public health surveys, and the health status of the study population is probably overestimated. On the other hand, partial non-response in any of the EQ5D dimensions assessed was very low, thus, preventing any further underestimation. Moreover, the low response rate also raises the question of power in the analysis, especially the sub-group analysis. To address this issue, a post-hoc power analysis was calculated based on a MCI of 0.082, which has previously been identified in HRQoL studies [26, 27]. The study results show a standard deviation of 0.15 in the reference group and 0.25 for the injury group. According to the post-hoc power calculation, between 50 and 200 persons were required in each comparison group, giving a power of 80% at 5% significance. In the results where we have a larger difference between the groups than 0.082, the power is above 80%; however, sometimes is it less, e.g., in the case of differences between MAIS1 and MAIS3+ for women aged 7–17 years, the difference is 0.22, which gives a power of 65%. Ideally, the number of participants would have been larger in each sub-group, giving the opportunity to always detect statistically significant differences as small as 0.082. Thus, adding additional participants would probably generate more statistically significant results; however, it is plausible that this would not change the conclusions of the results.

Although the current study has the above-mentioned limitations, the generalizability of the results should be sound considering an adult injury population as we have not been able to identify a systematic bias regarding the non-responses in the different groups and injury severities included. However, the results might only be transferable to adult injury populations who seek emergency care.

Moreover, we also need to consider the skewness of the data in regard to the analysis. Although we did apply different variance stabilizing methods, the skewness was not corrected. With regard to the skewness of the data, it is important to remember that HRQoL data in injury populations are naturally negatively skewed due to a large number of individuals who report a high HRQoL; hence, the mean of the population is lower than the mode. However, when we compared the EQ5D mean scores with the ones published by Sun et al. [28] (with a representative sample of 57,009 citizens from Stockholm; response rate of 61%), the EQ5D mean scores for the current study population were slightly lower (0.05–0.06 points) compared with the mean scores attained by Sun et al. [28]. Furthermore, when comparing the current mean scores with another Swedish study conducted by Burström et al. [15], who used the EQ5D, the mean scores of the two studies are even more similar, although Burström et al. published their data 9 years before the data collection for this study. These comparisons suggest that the reference group used in this study is likely to be representative of the target population. The response rate analysis is presented in more detail in Monárrez-Espino et al. [13].

## Conclusion

The results show that even relatively minor road traffic injuries can lead to a significant impact on HRQoL, especially for women, compared to the non-injured reference group. Moreover, in the current study, the inherent reduction of HRQoL over age was considered; hence, the results indicated that younger individuals have a larger difference from the reference group in HRQoL, independent of the injury severity, compared to the older individuals, due to the reduction of HRQoL with increased age. Furthermore, women’s low HRQoL may also be considered long lasting, since they had not returned to the reference group’s level of HRQoL, regardless of age and MAIS classification. An improved understanding of age and gender differences in HRQoL loss following an RTI is needed to better understand the long-term consequences of injuries, from a public health perspective.

## Electronic supplementary material

Below is the link to the electronic supplementary material.

Electronic supplementary material 1 (DOCX 21 kb)
