# Supplementary material for: Quality of life following road traffic injury: the impact of age and gender
Source: Qual Life Res. 2020 Jan 20;29(6):1587–96. doi: 10.1007/s11136-020-02427-3 (PMC7253518; doi:10.1007/s11136-020-02427-3)
Supplement: Supplementary file 1 — Electronic supplementary material 1 (DOCX 21 kb) [file 11136_2020_2427_MOESM1_ESM.docx]

Appendix 1. Numbers of injured body region by injury severity of the final sample after randomised stratified sample.

| Body region | MAIS  n (response rate %) | | | Total |
| --- | --- | --- | --- | --- |
|  | 1 | 2 | 3+ |  |
| Head | 200 (26) | 200 (39) | 200 (38) | 600 (35) |
| Cervical spine | 200 (39) | 200 (51) | 104 (38) | 504 (42) |
| Face | 200 (39) | 200 (37) | 55 (47) | 455 (39) |
| Upper extremities | 200 (36) | 200 (50) | 67 (37) | 467 (42) |
| Lower extremities and pelvis | 200 (32) | 200 (38) | 200 (41) | 600 (37) |
| Thorax | 200 (37) | 200 (44) | 200 (40) | 600 (40) |
| Thoracic spine | 200 (34) | 200 (36) | 51 (44) | 451 (36) |
| Abdomen | 26 (20) | 125 (41) | 77 (43) | 228 (40) |
| Lumbar spine | 200 (29) | 200 (47) | 44 (40) | 444 (38) |
| External | 200 (27) | 200 (34) | 12 (23) | 412 (30) |
| Total | 1826 (33) | 1925 (41) | 1010 (40) | 4761 (38) |
